# *catena*-Poly[[bis­[(2-carboxy­benzoato-κ*O*)silver(I)](*Ag*—*Ag*)]bis­(μ-isonicotinic acid-κ^2^ *N*:*O*)]

**DOI:** 10.1107/S1600536808009586

**Published:** 2008-05-07

**Authors:** Xiao-Feng Li, Yan An, Yan-Sheng Yin

**Affiliations:** a Institute of Marine Materials and Engineering, Shanghai Maritime University, Shanghai 200135, People’s Republic of China

## Abstract

The title compound, [Ag(C_8_H_5_O_4_)(C_6_H_5_NO_2_)]_*n*_, contains one Ag^I^ atom, one phthalate ligand and one isonicotinic acid mol­ecule in the asymmetric unit. Each Ag atom is three-coordinated in a T-shaped geometry by two O atoms and one N atom from one phthalate ligand and two isonicotinic acid ligands. The isonicotinic acid ligand bridges two Ag atoms, forming a one-dimensional chain. Adjacent chains are linked by Ag—Ag inter­actions, leading to a double-chain. These double-chains are further linked *via* hydrogen bonds into a two-dimensional layer.

## Related literature

For related literature, see: He *et al.* (2007 ▶); Xie *et al.* (2005 ▶).
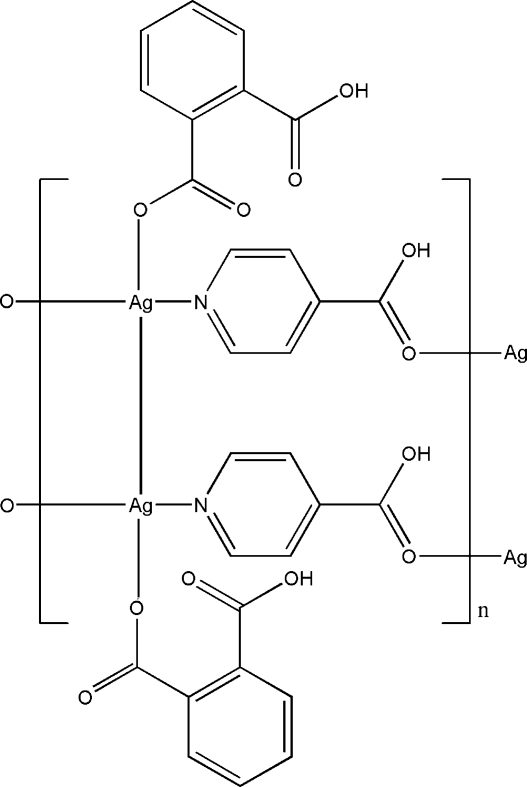


## Experimental

### 

#### Crystal data

[Ag(C_8_H_5_O_4_)(C_6_H_5_NO_2_)]
*M* *_r_* = 396.10
Monoclinic, 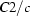

*a* = 13.540 (3) Å
*b* = 8.160 (2) Å
*c* = 24.223 (5) Å
β = 99.546 (15)°
*V* = 2639 (1) Å^3^
*Z* = 8
Mo *K*α radiation
μ = 1.56 mm^−1^
*T* = 293 (2) K
0.37 × 0.32 × 0.27 mm

#### Data collection

Siemens P4 four-circle diffractometer
Absorption correction: ψ scan (North *et al.*, 1968 ▶) *T*_min_ = 0.597, *T*_max_ = 0.680
3909 measured reflections
3037 independent reflections
1879 reflections with *I* > 2σ(*I*)
*R*_int_ = 0.034
3 standard reflections every 97 reflections intensity decay: 1.0%

#### Refinement

*R*[*F* ^2^ > 2σ(*F* ^2^)] = 0.042
*wR*(*F* ^2^) = 0.119
*S* = 1.00
3037 reflections
199 parameters
H-atom parameters constrained
Δρ_max_ = 0.99 e Å^−3^
Δρ_min_ = −0.71 e Å^−3^

### 

Data collection: *XSCANS* (Siemens, 1994 ▶); cell refinement: *XSCANS*; data reduction: *XSCANS*; program(s) used to solve structure: *SHELXS97* (Sheldrick, 2008 ▶); program(s) used to refine structure: *SHELXL97* (Sheldrick, 2008 ▶); molecular graphics: *X-SEED* (Barbour, 2001 ▶); software used to prepare material for publication: *publCIF* (Westrip, 2008 ▶).

## Supplementary Material

Crystal structure: contains datablocks I, global. DOI: 10.1107/S1600536808009586/hy2121sup1.cif

Structure factors: contains datablocks I. DOI: 10.1107/S1600536808009586/hy2121Isup2.hkl

Additional supplementary materials:  crystallographic information; 3D view; checkCIF report

## Figures and Tables

**Table:** 

Ag1—N1	2.179 (4)
Ag1—O3	2.185 (3)
Ag1—O2^i^	2.621 (3)
Ag1—Ag1^ii^	3.2123 (11)

**Table:** 

N1—Ag1—O3	164.57 (14)
N1—Ag1—O2^i^	93.52 (12)
O3—Ag1—O2^i^	101.74 (11)

Symmetry codes: (i) 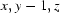; (ii) 
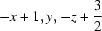
.

**Table 2:** Hydrogen-bond geometry (Å, °)

*D*—H⋯*A*	*D*—H	H⋯*A*	*D*⋯*A*	*D*—H⋯*A*
O1—H1*A*⋯O6^iii^	0.82	1.80	2.616 (5)	175
O5—H5*A*⋯O4	0.82	1.57	2.390 (5)	180

Symmetry code: (iii) 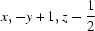.

**Fig. 1.**
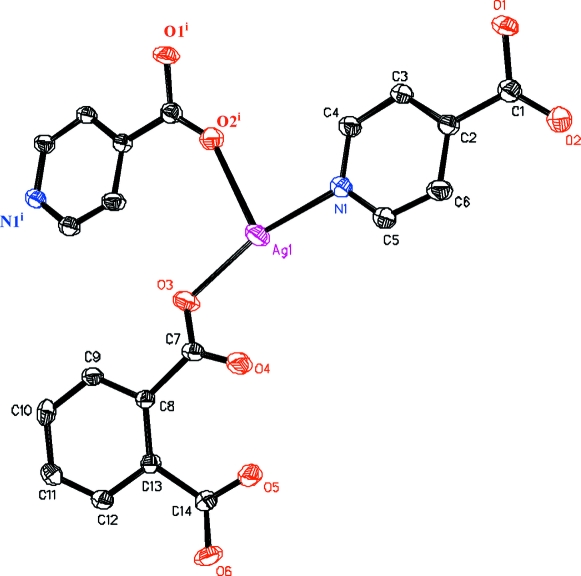
The coordination geometry of the Ag atom in the title compound. Displacement ellipsoids are drawn at the 30% probability level. [Symmetry code: (i) x, -1 + y, z.]

**Fig. 2.**
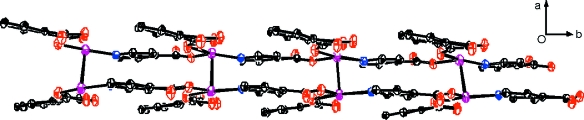
The one-dimensional double-chain connected by Ag···Ag interactions.

## supplementary crystallographic information

### Comment

Silver ion reacts with isonicotinic acid and imidazole under hydrothermal conditions to form [Ag_8_(in)_6_(NO_3_)_2_] and [Ag(in)(Hin)]_0.5_[Ag(in)] (Hin = isonicotinic acid) (Xie *et al.*, 2005). With phthalic acid in place of imidazole, the reaction yields the title compound.

In the title compound, the Ag^I^ atom is three-coordinated by two O atoms and one N atom from one phthalate ligand and two isonicotinic acid ligands in a T-like geometry, with an O—Ag—N bond angle being 164.57 (14)° (Fig. 1; Table 1), giving a chain structure. Furthermore, the adjacent chains are linked by Ag···Ag interactions (He *et al.*, 2007) to form a one-dimensional double-chain (Fig. 2). These double-chains are further linked *via* O—H···O hydrogen bonds (Table 2) into a two-dimensional layer. The hydrogen bonding interactions enhance the stability of the complex.

### Experimental

A mixture of Ag(NO_3_) (0.085 g, 0.5 mmol), isonicotinic acid (0.123 g, 1 mmol), phthalic acid (0.166 g, 1 mmol) and water (10 ml) was sealed in a 23 ml Teflon-lined reactor, which was heated at 473 K for 4 d and then cooled to room temperature at a rate of 5 K h^-1^ (yield 72%). Analysis calculated for C_14_H_10_AgNO_6_: C 42.45, H 2.54, N 3.54%; found: C 42.39, H 2.61, N 3.48%.

### Refinement

H atoms were positioned geometrically and refined as riding atoms, with C—H = 0.93 and O—H = 0.82Å and *U*_iso_(H) = 1.2*U*_eq_(C,*O*).

### Crystal data

**Table:**

[Ag(C_8_H_5_O_4_)(C_6_H_5_NO_2_)]	*F*_000_ = 1568
*M**_r_* = 396.10	*D*_x_ = 1.994 Mg m^−^^3^
Monoclinic, *C*2/*c*	Mo *K*α radiation λ = 0.71073 Å
Hall symbol: -C 2yc	Cell parameters from 28 reflections
*a* = 13.540 (3) Å	θ = 5.2–12.4º
*b* = 8.160 (2) Å	µ = 1.56 mm^−^^1^
*c* = 24.223 (5) Å	*T* = 293 (2) K
β = 99.546 (15)º	Block, purple
*V* = 2639 (1) Å^3^	0.37 × 0.32 × 0.27 mm
*Z* = 8	

### Data collection

**Table:**

Siemens P4 four-circle diffractometer	*R*_int_ = 0.034
Radiation source: medium-focus sealed tube	θ_max_ = 27.5º
Monochromator: graphite	θ_min_ = 1.7º
*T* = 293(2) K	*h* = −17→1
ω–2θ scans	*k* = −1→10
Absorption correction: ψ scan(North *et al.*, 1968)	*l* = −31→31
*T*_min_ = 0.597, *T*_max_ = 0.680	3 standard reflections
3909 measured reflections	every 97 reflections
3037 independent reflections	intensity decay: 1.0%
1879 reflections with *I* > 2σ(*I*)	

### Refinement

**Table:**

Refinement on *F*^2^	Secondary atom site location: difference Fourier map
Least-squares matrix: full	Hydrogen site location: inferred from neighbouring sites
*R*[*F*^2^ > 2σ(*F*^2^)] = 0.042	H-atom parameters constrained
*wR*(*F*^2^) = 0.119	*w* = 1/[σ^2^(*F*_o_^2^) + (0.0526*P*)^2^] where *P* = (*F*_o_^2^ + 2*F*_c_^2^)/3
*S* = 1.00	(Δ/σ)_max_ = 0.001
3037 reflections	Δρ_max_ = 0.99 e Å^−^^3^
199 parameters	Δρ_min_ = −0.71 e Å^−^^3^
Primary atom site location: structure-invariant direct methods	Extinction correction: none

### Fractional atomic coordinates and isotropic or equivalent isotropic displacement parameters (Å^2^)

**Table:**

	*x*	*y*	*z*	*U*_iso_*/*U*_eq_	
Ag1	0.40822 (3)	0.04719 (5)	0.698630 (15)	0.05417 (17)	
C1	0.4164 (4)	0.7535 (6)	0.58243 (19)	0.0405 (11)	
C2	0.4211 (3)	0.5872 (5)	0.60835 (17)	0.0347 (10)	
C3	0.4339 (4)	0.4453 (6)	0.57882 (17)	0.0397 (10)	
H3A	0.4417	0.4514	0.5415	0.048*	
C4	0.4348 (4)	0.2962 (6)	0.60478 (18)	0.0413 (11)	
H4A	0.4430	0.2020	0.5844	0.050*	
C5	0.4137 (4)	0.4183 (6)	0.68640 (19)	0.0442 (12)	
H5B	0.4070	0.4087	0.7239	0.053*	
C6	0.4119 (4)	0.5724 (6)	0.66396 (19)	0.0455 (12)	
H6A	0.4046	0.6644	0.6856	0.055*	
C7	0.3575 (4)	−0.0952 (6)	0.80056 (17)	0.0383 (11)	
C8	0.3373 (3)	−0.2215 (5)	0.84252 (16)	0.0307 (9)	
C9	0.3225 (3)	−0.3796 (6)	0.82196 (17)	0.0368 (10)	
H9A	0.3240	−0.3975	0.7842	0.044*	
C10	0.3056 (4)	−0.5119 (6)	0.8547 (2)	0.0437 (11)	
H10A	0.2958	−0.6164	0.8394	0.052*	
C11	0.3036 (4)	−0.4842 (6)	0.9109 (2)	0.0453 (12)	
H11A	0.2927	−0.5711	0.9340	0.054*	
C12	0.3177 (4)	−0.3304 (6)	0.93258 (18)	0.0403 (11)	
H12A	0.3159	−0.3145	0.9704	0.048*	
C13	0.3346 (3)	−0.1963 (5)	0.89994 (17)	0.0321 (9)	
C14	0.3497 (4)	−0.0355 (6)	0.93203 (18)	0.0411 (11)	
N1	0.4245 (3)	0.2820 (5)	0.65827 (15)	0.0414 (9)	
O1	0.3992 (3)	0.7496 (5)	0.52779 (12)	0.0626 (11)	
H1A	0.3896	0.8431	0.5156	0.094*	
O2	0.4267 (3)	0.8778 (4)	0.60899 (13)	0.0513 (9)	
O3	0.3817 (3)	−0.1459 (4)	0.75691 (13)	0.0579 (10)	
O4	0.3483 (3)	0.0565 (4)	0.80925 (14)	0.0619 (11)	
O5	0.3438 (3)	0.1015 (4)	0.90631 (14)	0.0579 (10)	
H5A	0.3454	0.0858	0.8730	0.087*	
O6	0.3672 (3)	−0.0408 (4)	0.98320 (13)	0.0641 (11)	

### Atomic displacement parameters (Å^2^)

**Table:**

	*U*^11^	*U*^22^	*U*^33^	*U*^12^	*U*^13^	*U*^23^
Ag1	0.0797 (3)	0.0432 (2)	0.0430 (2)	0.0001 (2)	0.02032 (19)	0.01176 (18)
C1	0.046 (3)	0.038 (3)	0.037 (2)	0.002 (2)	0.007 (2)	0.002 (2)
C2	0.037 (2)	0.037 (2)	0.030 (2)	−0.001 (2)	0.0055 (18)	0.0028 (18)
C3	0.055 (3)	0.036 (2)	0.028 (2)	−0.003 (2)	0.008 (2)	0.0023 (19)
C4	0.055 (3)	0.037 (2)	0.034 (2)	0.000 (2)	0.012 (2)	−0.0002 (19)
C5	0.061 (3)	0.039 (3)	0.034 (2)	−0.002 (2)	0.012 (2)	0.0022 (19)
C6	0.065 (3)	0.042 (3)	0.031 (2)	0.005 (3)	0.014 (2)	−0.002 (2)
C7	0.046 (3)	0.042 (3)	0.027 (2)	−0.001 (2)	0.0060 (19)	0.0026 (19)
C8	0.038 (2)	0.028 (2)	0.0250 (18)	−0.0007 (19)	0.0035 (17)	0.0007 (17)
C9	0.050 (3)	0.034 (2)	0.0278 (19)	−0.001 (2)	0.0098 (19)	−0.0021 (18)
C10	0.045 (3)	0.027 (2)	0.057 (3)	−0.003 (2)	0.004 (2)	−0.001 (2)
C11	0.058 (3)	0.037 (3)	0.042 (2)	−0.005 (2)	0.009 (2)	0.010 (2)
C12	0.051 (3)	0.040 (3)	0.030 (2)	−0.003 (2)	0.007 (2)	0.0033 (19)
C13	0.038 (2)	0.028 (2)	0.031 (2)	0.002 (2)	0.0056 (18)	0.0000 (17)
C14	0.053 (3)	0.036 (3)	0.035 (2)	−0.004 (2)	0.012 (2)	−0.006 (2)
N1	0.053 (2)	0.038 (2)	0.0349 (18)	−0.0020 (19)	0.0101 (18)	0.0034 (17)
O1	0.112 (3)	0.042 (2)	0.0295 (16)	−0.002 (2)	−0.0001 (19)	0.0104 (15)
O2	0.075 (3)	0.0364 (18)	0.0425 (18)	0.0043 (19)	0.0082 (17)	0.0005 (16)
O3	0.101 (3)	0.0417 (19)	0.0379 (17)	0.004 (2)	0.0313 (19)	0.0044 (16)
O4	0.116 (3)	0.0339 (18)	0.0379 (17)	−0.003 (2)	0.019 (2)	0.0053 (15)
O5	0.107 (3)	0.0303 (17)	0.0380 (17)	−0.006 (2)	0.017 (2)	−0.0037 (15)
O6	0.117 (3)	0.047 (2)	0.0278 (15)	−0.004 (2)	0.0086 (19)	−0.0078 (16)

### Geometric parameters (Å, °)

**Table:**

Ag1—N1	2.179 (4)	C7—O4	1.265 (6)
Ag1—O3	2.185 (3)	C7—C8	1.504 (6)
Ag1—O2^i^	2.621 (3)	C8—C9	1.385 (6)
Ag1—Ag1^ii^	3.2123 (11)	C8—C13	1.412 (5)
C1—O2	1.197 (6)	C9—C10	1.380 (6)
C1—O1	1.306 (5)	C9—H9A	0.9300
C1—C2	1.492 (6)	C10—C11	1.387 (7)
C2—C6	1.379 (6)	C10—H10A	0.9300
C2—C3	1.386 (6)	C11—C12	1.361 (7)
C3—C4	1.368 (6)	C11—H11A	0.9300
C3—H3A	0.9300	C12—C13	1.391 (6)
C4—N1	1.331 (5)	C12—H12A	0.9300
C4—H4A	0.9300	C13—C14	1.521 (6)
C5—N1	1.325 (6)	C14—O6	1.223 (5)
C5—C6	1.368 (7)	C14—O5	1.276 (6)
C5—H5B	0.9300	O1—H1A	0.8200
C6—H6A	0.9300	O5—H5A	0.8200
C7—O3	1.229 (5)		
			
N1—Ag1—O3	164.57 (14)	C9—C8—C7	115.3 (3)
N1—Ag1—O2^i^	93.52 (12)	C13—C8—C7	127.1 (4)
O3—Ag1—O2^i^	101.74 (11)	C10—C9—C8	123.4 (4)
N1—Ag1—Ag1^ii^	102.98 (11)	C10—C9—H9A	118.3
O3—Ag1—Ag1^ii^	71.85 (11)	C8—C9—H9A	118.3
O2—C1—O1	123.4 (4)	C9—C10—C11	117.9 (4)
O2—C1—C2	123.5 (4)	C9—C10—H10A	121.0
O1—C1—C2	113.1 (4)	C11—C10—H10A	121.0
C6—C2—C3	118.0 (4)	C12—C11—C10	120.3 (4)
C6—C2—C1	119.1 (4)	C12—C11—H11A	119.8
C3—C2—C1	122.9 (4)	C10—C11—H11A	119.8
C4—C3—C2	119.8 (4)	C11—C12—C13	122.2 (4)
C4—C3—H3A	120.1	C11—C12—H12A	118.9
C2—C3—H3A	120.1	C13—C12—H12A	118.9
N1—C4—C3	122.0 (4)	C12—C13—C8	118.6 (4)
N1—C4—H4A	119.0	C12—C13—C14	114.1 (4)
C3—C4—H4A	119.0	C8—C13—C14	127.3 (4)
N1—C5—C6	124.3 (4)	O6—C14—O5	120.8 (4)
N1—C5—H5B	117.9	O6—C14—C13	118.3 (4)
C6—C5—H5B	117.9	O5—C14—C13	120.9 (4)
C5—C6—C2	118.0 (5)	C5—N1—C4	117.8 (4)
C5—C6—H6A	121.0	C5—N1—Ag1	118.6 (3)
C2—C6—H6A	121.0	C4—N1—Ag1	123.3 (3)
O3—C7—O4	121.4 (4)	C1—O1—H1A	109.5
O3—C7—C8	117.0 (4)	C7—O3—Ag1	114.1 (3)
O4—C7—C8	121.6 (4)	C14—O5—H5A	109.5
C9—C8—C13	117.6 (4)		
			
O2—C1—C2—C6	16.8 (8)	C9—C8—C13—C12	0.2 (7)
O1—C1—C2—C6	−162.7 (4)	C7—C8—C13—C12	−178.0 (4)
O2—C1—C2—C3	−163.7 (5)	C9—C8—C13—C14	179.3 (4)
O1—C1—C2—C3	16.8 (7)	C7—C8—C13—C14	1.1 (8)
C6—C2—C3—C4	1.3 (7)	C12—C13—C14—O6	15.3 (7)
C1—C2—C3—C4	−178.2 (5)	C8—C13—C14—O6	−163.8 (5)
C2—C3—C4—N1	−0.5 (8)	C12—C13—C14—O5	−164.4 (5)
N1—C5—C6—C2	0.4 (8)	C8—C13—C14—O5	16.5 (8)
C3—C2—C6—C5	−1.3 (7)	C6—C5—N1—C4	0.4 (8)
C1—C2—C6—C5	178.3 (5)	C6—C5—N1—Ag1	−173.0 (4)
O3—C7—C8—C9	−16.1 (6)	C3—C4—N1—C5	−0.4 (7)
O4—C7—C8—C9	162.6 (5)	C3—C4—N1—Ag1	172.6 (4)
O3—C7—C8—C13	162.1 (5)	O3—Ag1—N1—C5	4.1 (8)
O4—C7—C8—C13	−19.2 (8)	Ag1^ii^—Ag1—N1—C5	−64.4 (4)
C13—C8—C9—C10	−0.1 (7)	O3—Ag1—N1—C4	−168.9 (5)
C7—C8—C9—C10	178.3 (4)	Ag1^ii^—Ag1—N1—C4	122.6 (4)
C8—C9—C10—C11	−0.1 (8)	O4—C7—O3—Ag1	0.9 (6)
C9—C10—C11—C12	0.3 (8)	C8—C7—O3—Ag1	179.6 (3)
C10—C11—C12—C13	−0.2 (8)	N1—Ag1—O3—C7	0.2 (8)
C11—C12—C13—C8	−0.1 (7)	Ag1^ii^—Ag1—O3—C7	72.7 (4)
C11—C12—C13—C14	−179.3 (5)		

Symmetry codes: (i) *x*, *y*−1, *z*; (ii) −*x*+1, *y*, −*z*+3/2.

### Hydrogen-bond geometry (Å, °)

**Table:**

*D*—H···*A*	*D*—H	H···*A*	*D*···*A*	*D*—H···*A*
O1—H1A···O6^iii^	0.82	1.80	2.616 (5)	175
O5—H5A···O4	0.82	1.57	2.390 (5)	180

Symmetry codes: (iii) *x*, −*y*+1, *z*−1/2.
